# Blunt Traumatic Aortic Injury Treated with Endovascular Aortic Repair: Does Age Influence the Outcome?

**DOI:** 10.3390/jcm14030776

**Published:** 2025-01-24

**Authors:** Maximilian Lutz, David Wippel, Alexander Loizides, Malik Galijasevic, Laura Schönherr, Elke R. Gizewski, Sabine Wipper, Martin Freund, Florian K. Enzmann

**Affiliations:** 1 Department of Radiology, Medical University of Innsbruck, 6020 Innsbruck, Austria; maximilian.lutz@i-med.ac.at (M.L.); malik.galijasevic@i-med.ac.at (M.G.); elke.gizewski@i-med.ac.at (E.R.G.); martin.freund@i-med.ac.at (M.F.); 2 Department of Vascular Surgery, Medical University of Innsbruck, 6020 Innsbruck, Austria; david.wippel@i-med.ac.at (D.W.); laura.schoenherr@i-med.ac.at (L.S.); sabine.wipper@i-med.ac.at (S.W.); florian.enzmann@i-med.ac.at (F.K.E.)

**Keywords:** BTAI, blunt aortic injury, aortic trauma, TEVAR

## Abstract

**Background**: Blunt traumatic aortic injury (BTAI) is the second most common cause of death following blunt trauma, and it can affect people of all ages. The aim of this study was to evaluate age-related differences in outcomes among patients undergoing thoracic endovascular aortic repair (TEVAR) for BTAI. **Methods**: All patients treated with TEVAR for BTAI at a tertiary care center in Europe between 2005 and 2023 were included in this study. All clinical and imaging data were collected and analyzed retrospectively. **Results**: A total of 70 patients with a median age of 43 years were included, and 89% were male. Older patients had significantly higher American Society of Anesthesiologists (ASA) physical status classification scores (*p* < 0.001) compared to younger patients. All age groups (<18, 18–40, 41–65, and >65) exhibited low to borderline low initial hemoglobin levels with a further decline over time (*p* = 0.063, *p* < 0.001, *p* < 0.001, and *p* = 0.018, respectively). Age groups were comparable regarding injury mechanism, Injury Severity Score (ISS), concomitant injuries and postoperative complications. The age-independent ISS showed a moderate to strong correlation to the length of intensive care unit stay (r = 0.594, *p* < 0.001). Total in-hospital mortality was 6% and none was from aortic-related complications. There was a generally high rate of loss of follow-up (59%). **Conclusions**: Although older patients presented worse ASA scores in comparison to younger patients, no significant differences regarding postoperative morbidity/mortality were noted. These findings imply that patient age and preinjury physical status might not substantially influence outcomes when treating BTAI with TEVAR.

## 1. Introduction

Blunt traumatic aortic injury (BTAI) is a rare but deadly complication of thoracic trauma. Although occurring only in about 1% of trauma cases, it accounts for the second most common cause of death following blunt trauma [1,2,3,4]. A large proportion (up to 80%) of patients suffering from BTAI die before receiving sufficient treatment [5]. BTAI most commonly results from sudden deceleration, with motor vehicle accidents being the predominant mechanism described [2,3,5,6,7]. The age distribution of BTAI is broad, with a mean age of 35 to 45 years, and there is a male predominance [1,2,3,7].

Azizzadeh et al. [8] published a classification system for traumatic aortic injuries, which consists of four grades: grade I (intimal tear), grade II (intramural hematoma), grade III (pseudoaneurysm), and grade IV (rupture). With the evolution of thoracic endovascular aortic repair (TEVAR) and the advancement of devices over the last decades, endovascular therapy has become the standard of care in favor of open surgery, resulting in lower mortality rates [9,10,11,12]. The European Society of Vascular Surgery (ESVS) guidelines on the management of descending thoracic aorta diseases from 2017 recommend TEVAR as a first-line treatment for BTAI in patients with suitable anatomy with the exception of minimal aortic injuries that present with an intimal tear, which may be managed conservatively [6]. This recommendation was reaffirmed in the recently published ESVS Clinical Practice Guidelines on the Management of Vascular Trauma, which advocate TEVAR as the preferred treatment strategy for traumatic pseudoaneurysms, traumatic abnormal external wall contours, and free ruptures [13]. Regarding pseudoaneurysms and abnormal external wall contours, the guidelines further recommend intervention within 24 h if high-risk factors, such as a large mediastinal hematoma, left-sided hemothorax, aortic coarctation, a large size of the pseudoaneurysm, systolic blood pressure < 90 mmHg or traumatic brain injury, are present, while otherwise delayed TEVAR should be performed [13].

The aim of this study was to review and analyze data from patients treated for BTAI at our center, focusing on age-related differences in preintervention comorbidities, procedural characteristics, and clinical outcomes. We hypothesized that outcomes would differ significantly across age groups, reflecting the influence of age on treatment efficacy and recovery.

## 2. Materials and Methods

### 2.1. Study Design

A retrospective analysis of all patients who received TEVAR after being diagnosed with BTAI from 2005 to 2023 at a tertiary care center in Europe was performed. Ethical approval for this study was obtained from the local ethics committee of our university (EK Nr. 1333/2022).

Patient-specific data were reviewed using the local electronic patient record system “KIS” (Cerner Corporation, North Kansas City, MO, USA), while clinical imaging data were analyzed using the DeepUnity Diagnost Picture Archiving and Communication System (version 2.0.2.2, Dedalus Healthcare Group, Milan, Italy). All measurements were conducted on preintervention CT datasets by a resident with two years of experience and a consultant with 17 years of expertise. Study data were collected and managed using REDCap (version 14.3.12, Vanderbilt University, Nashville, TN, USA), hosted at our local university [14,15]. In addition to analyzing existing data, the Injury Severity Score (ISS), based on the Abbreviated Injury Scale, was calculated retrospectively for each patient [16]. The aortic zones of injury, as well as the landing zones, were determined according to the zones of attachment, described by Fillinger et al. [17].

### 2.2. Statistical Analysis

Statistical analysis was performed using R (R Core Team v. 3.6.1). The Shapiro–Wilk test and one-sample Kolmogorov–Smirnov test were used to assess the normality of data. For the analysis of more than two groups in not normally distributed data, the Kruskal–Wallis test was used. For the analysis of more than two groups in normally distributed data ANOVA was used. The ANOVA compatibility was tested with the Laven test. *p*-values < 0.05 were considered statistically significant. For statistically significant results, Dunn’s post hoc test and pairwise-t post hoc tests were used. The results are presented using boxplots and tables. For binary/categorical data, the Chi-square test of independence was used. For binary data with small sample numbers, Fisher’s exact test for count data was used. For statistically significant results, standardized residuals were extracted and presented in a table. For the standardized residuals, adjusted alpha values and critical values were used. Changes in hemoglobin levels between two time points were analyzed separately for each age group using the Wilcoxon Signed-Rank Test, as the data were not normally distributed. Associations between clinical parameters and outcomes were evaluated. Continuous and ordinal variables were analyzed using Spearman’s rank correlation. Binary variables, such as concomitant injuries and postoperative outcomes, were analyzed using Fisher’s exact test or Chi-squared test, depending on cell sizes. Associations between binary predictors and continuous outcomes were explored using the Mann–Whitney U Test. The results are presented as correlation coefficients (r) or odds ratios with 95% confidence intervals. For significant and non-significant correlations, a post hoc power analysis was performed using the observed correlation coefficients and the sample size to assess whether the study was adequately powered. The values are presented as mean ± standard deviation (SD) for normally distributed data and as median with interquartile range (IQR) for not normally distributed data. For highly skewed data, percentile quartiles are presented.

For statistical analyses, the following subgroups were defined: <18 years (G1), 18–40 years (G2), 41–65 years (G3), and >65 years (G4).

## 3. Results

### 3.1. Patient Characteristics

A total of 70 patients with BTAI were treated with TEVAR after being admitted to a tertiary care center in Europe. The median age was 43 years, ranging from 13 to 76 years, and 62 of the 70 participants were male. Most patients did not take any premedication (54/70), and the most common underlying health condition was hypertension (17/70). A detailed overview of the basic demographics is presented in Table 1.

**Figure 1:**
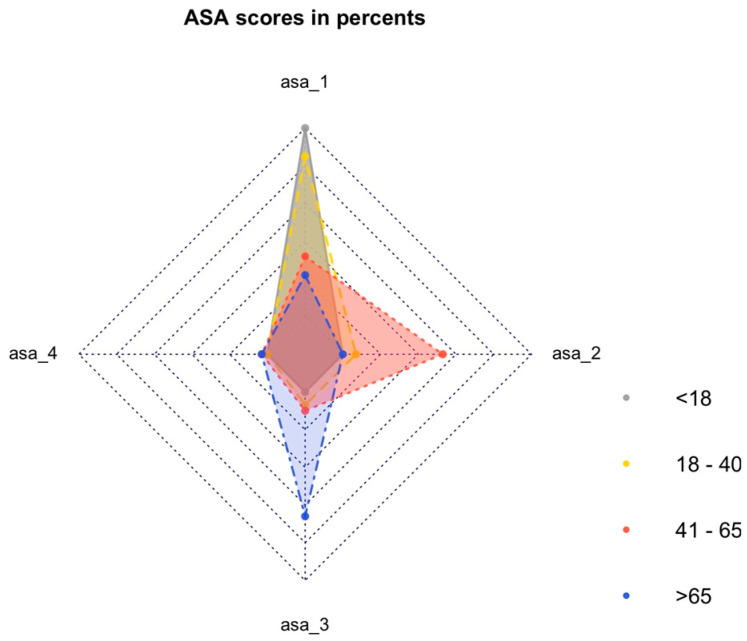
ASA classification by percentage in different age groups.

A significant difference was observed in the American Society of Anesthesiologists (ASA) physical status classification score before injury, with older patients having higher ASA scores compared to younger patients (*p* < 0.001) (Figure 1).

Patients > 65 years had significantly more cases of coronary artery disease (CAD) (*p* = 0.029) and received significantly more premedication with acetylsalicylic acid (*p* = 0.011) and beta-blockers (*p* = 0.003) compared to the other age groups.

### 3.2. Trauma Characteristics and Aortic Pathology

The most common underlying trauma mechanisms were skiing accidents (25/70), followed by motor vehicle accidents (23/70). All patients suffered from concomitant injuries. The median ISS was 25 with numbers ranging from 16 to 75. Most patients presented with a BTAI grade III injury (60/70), followed by grade II (7/70) and grade IV (3/70). No BTAI grade I injury was treated endovascularly. The most common aortic zones of injury were zone 3, with 43 cases, and zone 4, with 17 cases, while zone 2 and zone 5 were less common (6/70 and 4/70, respectively). There was no significant difference in the zones of injury between the subgroups (*p* = 0.957). Data regarding trauma characteristics and aortic pathology specifications are represented in Table 2.

**Figure 2:**
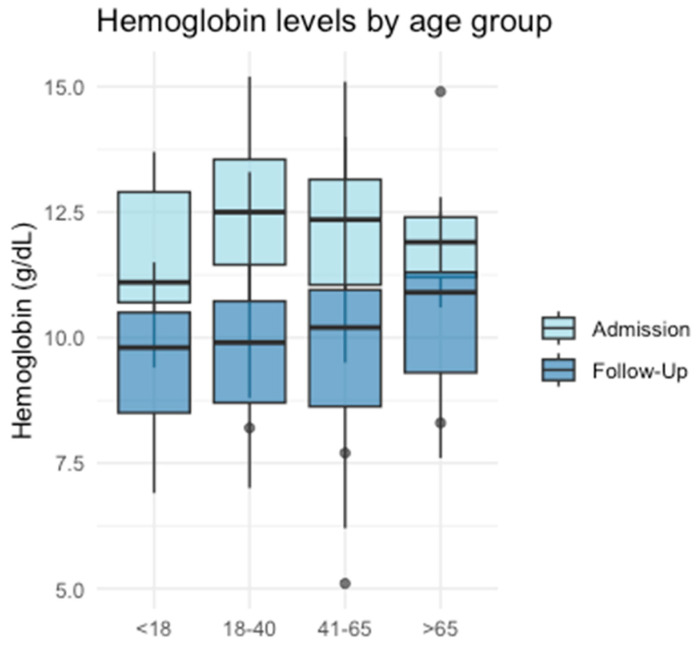
Hemoglobin levels in different age groups.

When comparing initial hemoglobin levels with those measured at the first follow-up, a statistically significant decline was observed in G2 (*p* < 0.001), G3 (*p* < 0.001), and G4 (*p* = 0.018). Although a decrease was also noted in G1, this did not reach statistical significance (*p* = 0.063) (Figure 2).

**Figure 3:**
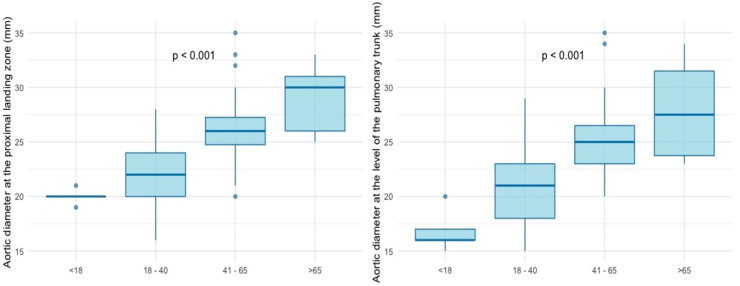
Aortic diameters at the proximal landing zone and at the level of the pulmonary trunk.

Younger patients had significantly smaller aortic diameters at the proximal landing zone as well as at the level of the pulmonary trunk, with diameters gradually increasing with each subgroup (*p* < 0.001, respectively) (Figure 3). After Bonferroni correction, the strongest statistical significance was observed between G2 and G3 as well as G2 and G4.

### 3.3. Endovascular Intervention

The vast majority (57/70) of patients were treated the same day that the accident happened (4 patients from G1, 24 from G2, 23 from G3, and 6 from G4), and no significant differences were noted between the different age groups (*p* = 0.466).

According to the increasing aortic diameters with age, the diameters of the implanted stents were significantly increasing within the age groups as well (*p* < 0.001). In seven of the 70 cases, a second stent had to be implanted (in two patients from G2, three from G3, two from G4). A single uncovered stent was implanted to treat a complex post-traumatic dissection, which had already caused severe ischemia of the visceral organs and ultimately resulted in death shortly thereafter. In contrast, all other devices used were covered stent grafts. The following stents were implanted: Valiant (34/77) and Talent (12/77) (Medtronic PLC, Dublin, Ireland), TAG (20/77) and Excluder (1/70) (W.L. Gore & Associates, Delaware, USA), Relay Pro (3/77), Relay NBS (1/77) and Relay Plus (2/77) (Terumo Aortic/Bolton Medical Inc., Sunrise, FL, USA), and Sinus XL (1/70) (optimed Medizinische Instrumente GmbH, Ettlingen, Germany). In three instances, there were missing data regarding the implanted model.

The proximal TEVAR landing zone was in zone 2 in 48 patients, covering the left subclavian artery (LSA), and in zone 3 in 17 patients. More distal proximal landing zones were less frequent. No significant differences were noted in the proximal landing zones (*p* = 0.131). Only two patients with a proximal landing zone 2 received prior endovascular embolization of the LSA.

Complications during the intervention occurred in five patients, four of which were from G3. There was one case of a short-distance dissection at the right common iliac artery, which had no therapeutic consequences. Another patient suffered persistent bleeding in the right groin after percutaneous access, which had to be treated surgically. One patient showed a “bird-beak” configuration at the proximal landing zone, which was associated with the kinking of the graft. In one instance, there was a distal dislocation of the stent graft while at deployment, so a second one had to be inserted. In the fifth complication, which affected a patient from G2, the stent graft could not be placed over the main aortic pathology, so it was inserted more distally to cover another small pseudoaneurysm. A second stent was implanted to cover the main aortic lesion.

In addition to gradually increasing the diameters of the implanted stents with age (*p* < 0.001), the subgroup analysis showed no significant differences between the different age groups.

### 3.4. Short-Term Outcome

Four out of the 70 patients died during their hospital stay: two because of multiorgan failure, one because of cardiac failure, and one because of brainstem herniation. Two of the deaths occurred within one day after admission, one on day 10 and one on day 49.

The median postinterventional stay at the intensive care unit (ICU) was nine days, ranging from zero to a maximum of 90 days. The median overall hospital stay was 15.5 days. Forty-six patients were transported to a hospital near their hometown for further rehabilitation after a median of 17.5 (10–19) days, while 20 patients were discharged directly to their home after a median of 14 (8–25) days.

No significant differences were observed between the individual groups regarding postinterventional ICU stay. However, a correlation analysis independent of group allocation revealed a moderate to strong positive correlation between the ISS and ICU stay (r = 0.594, *p* < 0.001).

Regarding the 30-day postoperative morbidity, four patients had a present endoleak at the first postinterventional CT, which were all classified as type IIa. Despite covering the LSA in 48 patients, none presented with ischemic symptoms of their left upper extremity. Five patients suffered from a major stroke; however there was correlation to accident-related head trauma in four of these cases. In one patient whose LSA was covered, the stroke was most likely related to the TEVAR. Six patients suffered from permanent paraplegia, five of which had a causal connection to the initial trauma. In the remaining case, the exact cause of paraplegia could not be conclusively determined, raising the possibility of an association with TEVAR. A detailed overview of the 30-day morbidity is represented in Table 3.

There was a total of seven secondary interventions related to the TEVAR, which were performed during the same hospital stay. In two patients, a second fluoroscopy was performed to treat a type IIa endoleak. In the first case, balloon dilatation was attempted to treat the endoleak but proved unsuccessful (G1). In the other case, the endoleak was successfully resolved through the implantation of two Amplatzer Vascular Plugs II (Abbott Medical, Plymouth, ML, USA). Furthermore, this patient developed ischemic symptoms in the left upper extremity, so a carotid-subclavian bypass was chosen as a treatment strategy (G4). Another patient received a carotid-subclavian bypass, this time to prevent further malperfusion after initial paraplegia (G2). Two patients received a secondary intervention at the groin, one in order to treat a pseudoaneurysm (G4), and the other one due to acute bleeding (G3). One patient (G2) was immediately transferred to an operating room following endovascular repair for surgical management of trauma-associated abdominal organ injuries. During the repair of a diaphragmatic rupture, arterial bleeding from the left hemithorax was noted, and the patient became hemodynamically unstable shortly thereafter. Subsequent intraoperative evaluation revealed a complete rupture of the aorta at the isthmus. Consequently, the stent graft was excised, and a Hemashield vascular prosthesis was implanted.

With regard to the short-term outcome, there were no significant differences between the subgroups.

### 3.5. Follow-Up Data

Of the 66 surviving patients, 27 had at least one follow-up after discharge (2 patients from G1, 13 from G2, 10 from G3, and 2 from G4), 25 of whom were male. The median duration between the intervention and the first follow-up was five months. Computed tomography angiography was performed in 23 cases, and magnetic resonance angiography was performed in four cases.

Four of these patients presented with significant changes since discharge. The patient with the “bird-beak” configuration (G3) was diagnosed with a newly occurred type IIa endoleak. Furthermore, there was evidence of an in-stent thrombus. Since the patient was completely asymptomatic, an interdisciplinary conference decided on a conservative approach, which resulted in regular follow-ups. Another patient showed an in-stent thrombus, which was managed conservatively as well (G3). Two patients who had their LSA covered presented with newly occurred ischemic symptoms of their left upper extremity (weakness (G2)/dysaesthesia (G2)).

Seventeen out of the twenty-seven patients had at least one additional follow-up. The median follow-up period for these individuals was 95 months, ranging from 11 to a maximum of 199 months. All patients received further imaging. The patient with the conservatively treated type IIa endoleak showed minimal growth of the apposition thrombus, while the endoleak did not progress over time. All other patients showed no new changes related to their TEVAR.

No significant differences between the subgroups regarding their follow-up regime were noted.

## 4. Discussion

Although BTAI is a potentially fatal pathology, the available evidence of treatment strategies is scarce. This study provides an overview of our experience in treating patients with TEVAR after BTAI, and represents one of the largest single-center studies on this topic. Our findings imply that preoperative health status may not be a significant factor in outcomes for BTAI patients treated with TEVAR.

There was no reporting of the preintervention ASA score in other publications. The ASA score classifies patients regarding their preoperative overall health status [18,19]. Our analyses showed significant differences between the ASA scorings of the different age groups. All underaged patients had an ASA score of 1. Patients from G3 had an ASA score of 2, and patients from G4 had an ASA score of 3 more frequently than other groups. In general, the ASA scores increased with age, corresponding to a higher perioperative risk and a weaker physical status in older patients.

Patients > 65 years had significantly more preexisting CAD and received significantly more premedication with acetylsalicylic acid as well as beta-blockers. This is in accordance with the literature since the prevalence of CAD increases with age, and both drugs are considered substantial treatment options for this disease [20].

As the study center is located in a winter sports region, most patients suffered from skiing accidents. In the literature, there are only a few reports about aortic injuries after skiing accidents, while the overall most common cause of BTAI is motor vehicle accidents [3,6,7,21,22,23]. The median ISS in our cohort was 25, with an IQR of 15, which is lower than typical values in the literature [7,24,25]. This could be due to the composition of our study cohort, since patients suffering from BTAI after skiing accidents show lower ISS and fewer concomitant injuries when compared to motor vehicle accidents [26]. There were no significant differences in the ISS as well as in the distribution of concomitant injuries between the different age groups, suggesting that the severity of the traumatic injury was similar across all ages.

When analyzing the data irrespective of age groups, a moderate to strong positive correlation was observed between the ISS and the post-intervention ICU stay (r = 0.594, *p* < 0.001). This finding could indicate that short-term outcomes are influenced by the initial severity of trauma. For patients with BTAI, it has been demonstrated that the ISS is associated with increased mortality [3,27]. Additionally, multiple studies have shown that the ISS correlates, in general, with both mortality and poor long-term outcomes in trauma patients [28,29].

Consistent with the traumatic events, all age groups exhibited reduced or borderline low initial hemoglobin levels, primarily attributable to hemorrhages associated with the trauma. The subsequent decline in hemoglobin levels over time can be interpreted as indicative of ongoing active bleeding. This decrease was statistically significant in all groups except G1, although a corresponding trend was observed in this subgroup as well. These findings align with the existing literature, which has demonstrated that low hemoglobin levels and a decline in hemoglobin over time can predict significant bleeding in trauma patients [30]. Furthermore, low hemoglobin levels have been shown to correlate with an increased need for hemostatic interventions and higher mortality rates [31].

There was covering of the LSA in 48 out of the 70 patients (69%), which is higher in comparison to the literature [7,10,21,24]. Most aortic pathologies in our cohort occurred around the aortic isthmus and, in order to reach a proximal sealing zone of at least 2 cm, these injuries often required an overstenting of the LSA. To reduce the risk of postinterventional complications associated with LSA coverage, all patients underwent either a pre- or periinterventional evaluation of their vertebral arteries, performed using computed tomography angiography or during fluoroscopy.

The overall in-hospital mortality was low with 6% (4/70) compared to literature with reported rates up to 18%, even though a recent systematic review reported a comparable 30-day mortality with 5.0% (versus 4% (3/70) in our study) [1,3,7,9,21,25,32,33,34]. With respect to different age groups, there were no significant differences in-hospital mortality in our study cohort, even though older patients had significantly higher ASA scores. Skaga et al. [35] were able to show that the pre-injury ASA score was an independent predictor of mortality after trauma. The median ISS of their cohort was lower, indicating less severe trauma; yet the mortality rate was higher [35]. This could lead to the assumption that the ASA score is less significant for the risk assessment of patients with BTAI compared to general trauma patients.

Data regarding the influence of age when treating BTAI is rare. Alarhayem et al. [36] were able to show that an age > 65 years showed a higher adjusted mortality in patients who were treated with TEVAR after BTAI within 24 h of the accident. A study focusing on patients aged 65 or older after BTAI found that there was significantly lower mortality in patients treated with TEVAR compared to nonoperative management or open repair, even though the results still seem to be worse compared to the findings of age-independent results [37]. De Rango et al. [38] investigated the impact of age on survival after TEVAR and found significantly higher mortality rates in patients over the age of 75 in emergency settings as well as in acute rupture settings. Furthermore, another study demonstrated that age over 60 years is an independent predictor of mortality following BTAI and TEVAR [27]. In contrast to these studies, our findings suggest that patient age and preinjury physical status may not significantly affect outcomes in TEVAR-treated BTAI, with trauma severity appearing to be a more decisive factor.

In recent years, studies were able to show a reduction in mortality when TEVAR was delayed more than 24 h after the initial trauma [13,25,36]. All four patients in our cohort who died were treated within 24 h of the accident; however, a delay of intervention did not seem appropriate in one of these patients due to their overall condition.

Postoperative complications occurred in a similar way throughout all age groups without significant differences. The median postinterventional ICU stay was comparable between the subgroups as well.

In regard to the long-term durability of the TEVAR, there was no evidence of stent graft migration or infection. None of the complications that occurred during the follow-up required reintervention, and there was no record of death in any patient during the follow-up period. However, the high rate of loss of follow-up in our cohort requires a cautious interpretation of these findings. Several multicentric studies with larger sample sizes and more robust follow-up data have demonstrated the occurrence of late complications that may lead to reinterventions [32,33,34]. It is plausible that such patients were underrepresented in the follow-up in our cohort.

It has to be acknowledged that during this study period, all patients in our center, who presented with BTAI and the subsequent need for intervention, were treated with TEVAR, and there was no case of an open surgery. Therefore, there was no confounding bias in selecting a therapeutic strategy.

### Limitations

The main limitation of this study is its small cohort size, which could compromise the ability to discern subtle differences between the different age groups. Furthermore, there is an underrepresentation of patients under the age of 18 and over the age of 65.

Another limitation of this study is the high percentage of loss of follow-up, which is mainly attributed to the composition of the patient cohort. A large proportion of our study cohort were tourists, who did not attend follow-ups at our tertiary care center.

It should also be noted that due to the composition of the patient cohort, which includes many physically active people, there may be a disproportionately high proportion of fit older patients. This characteristic may influence the generalizability of the study findings, restricting their applicability to centers or regions with comparable patient demographics.

A further limitation of this study is the small number of events (n = 4) and the absence of aortic-related mortality, which prevents the use of advanced techniques such as Kaplan–Meier analysis or Cox regression. These methods require adequate event rates to produce stable hazard estimates and reliable subgroup comparisons. Consequently, applying them to our limited data would likely result in non-informative or unreliable outcomes.

## 5. Conclusions

BTAI remains a fatal complication of trauma, with TEVAR serving as a successful treatment strategy for patients of all ages. The in-hospital mortality in this study was low throughout all age groups and the occurred deaths were not related to aortic injury. Even though older patients showed worse preinterventional ASA scores, indicating a higher perioperative risk and a lower physical status, the outcome did not seem to be influenced by this.

## Figures and Tables

**Table 1:** Basic demographics.

	Cohort (n = 70)	<18 (n = 5)	18–40 (n = 28)	41–65 (n = 28)	>65 (n = 9)	*p*-Value
Age—y	43 (32)	14 (2)	28.5 (13)	52.5 (8)	69 (2)	
Male	62 (89)	4 (80)	25 (89)	26 (93)	7 (78)	0.58
BMI	25.7 (4)	25 (3)	24.7 (3)	24.8 (4)	26.3 (1)	0.54
Hypertension	17 (24)	0 (0)	3 (11)	9 (32)	5 (56)	0.015 ^1^
Dyslipoproteinemia	4 (6)	0 (0)	0 (0)	3 (9)	1 (11)	0.12
Diabetes	3 (4)	0 (0)	0 (0)	3 (11)	0 (0)	0.33
CAD	2 (3)	0 (0)	0 (0)	0 (0) ^2^	2 (22)	0.029
COPD	3 (4)	0 (0)	0 (0)	2 (7)	1 (11)	0.41
CHF	1 (1)	0 (0)	0 (0)	0 (0)	1 (11)	0.36
ASA score before injury (1/2/3/4)	40/17/11/2	5/0/0/0	24/2/2/0	9/15/3/1	2/0/6/1	<0.001
Acetylsalicylic acid therapy	5 (7)	0 (0)	0 (0)	2 (7)	3 (33)	0.011
Betablocker therapy	7 (10)	0 (0)	0 (0)	3 (11)	4 (44)	0.003

Data are presented as n (%), median (IQR), or absolute frequencies. BMI = body mass index; CAD = coronary artery disease; COPD = chronic obstructive pulmonary disease; CHF = congestive heart failure; ASA = American Society of Anesthesiologists. ^1^ No significance after post hoc tests and Bonferroni correction. ^2^ One case of unknown CAD prior to the injury.

**Table 2:** Trauma characteristics and aortic pathology data.

	Cohort (n = 70)	<18 (n = 5)	18–40 (n = 28)	41–65 (n = 28)	>65 (n = 9)	*p*-Value
Causes of injury						0.73
Skiing accidents	25 (36)	3 (60)	9 (32)	9 (32)	4 (44)	
Motor vehicle accidents	23 (33)	0 (0)	10 (36)	10 (36)	3 (33)	
Falls from height	13 (19)	0 (0)	6 (21)	6 (21)	1 (11)	
Pedestrian vs. motor vehicle	3 (4)	1 (20)	1 (4)	1 (4)	0 (0)	
Others	6 (9)	1 (20)	2 (7)	2 (7)	1 (11)	
Grade of aortic injury						0.22
II	7 (10)	0 (0)	2 (7)	2 (7)	3 (33)	
III	60 (86)	5 (100)	25 (89)	25 (89)	5 (56)	
IV	3 (4)	0 (0)	1 (4)	1 (4)	1 (11)	
Concomitant injuries						
Traumatic brain injury	22 (31)	1 (20)	11 (39)	8 (29)	2 (22)	0.73
Skull fracture	17 (24)	2 (40)	7 (25)	6 (21)	2 (22)	0.83
Cervical spine fracture	10 (14)	0 (0)	6 (21)	4 (14)	0 (0)	0.50
Thoracic spine fracture	15 (21)	1 (20)	3 (11)	9 (32)	2 (22)	0.24
Lumbar spine fracture	17 (24)	0 (0)	8 (29)	8 (29)	1 (11)	0.47
Rib fracture	52 (74)	3 (60)	18 (64)	23 (82)	8 (89)	0.29
Pelvic fracture	14 (20)	0 (0)	4 (14)	9 (32)	1 (11)	0.27
Extremity fracture	40 (57)	3 (60)	16 (57)	17 (61)	4 (44)	0.91
Mediastinal hemorrhage	57 (81)	5 (100)	24 (86)	23 (82)	5 (56)	0.19
Hemothorax	43 (61)	3 (60)	18 (64)	16 (57)	6 (67)	0.94
Lung injury	43 (61)	2 (40)	21 (75)	18 (64)	2 (22)	0.027 ^3^
Pneumothorax	36 (51)	1 (20)	15 (54)	16 (57)	4 (44)	0.50
Abdominal organ injury	35 (50)	3 (60)	15 (54)	13 (46)	4 (44)	0.90
ISS	25 (15)	24 (18)	25 (12)	24 (13)	25 (17)	0.74
Mean arterial pressure at admission in mmHg	84 (38)	95.5 (23.75)	73.5 (29)	91 (28)	88 (32.25)	0.16
Shock index at admission	0.75 (0.3)	0.55 (0.1)	0.8 (0.28)	0.75 (0.38)	0.6 (0.18)	0.03 ^4^
Hemoglobin at admission in g/dL	12.3 (2.28)	11.1 (2.2)	12.5 (2.1)	12.35 (2.1)	11.9 (1.2)	0.58
Hemoglobin within 6 h in g/dL	9.95 (2.4)	9.8 (2)	9.9 (2)	10.2 (2.3)	10.9 (2)	0.87

Data are presented as n (%) or median (IQR). ISS = Injury Severity Score. ^3^ No significance after post hoc tests and Bonferroni correction. ^4^ No significance after post hoc tests and Bonferroni correction

**Table 3:** Short-term outcome data.

	Cohort (n = 70)	<18 (n = 5)	18–40 (n = 28)	41–65 (n = 28)	>65 (n = 9)	*p*-Value
30-day morbidity						0.49
None	28 (40)	3 (60)	10 (36)	10 (36)	5 (56)	0.51
Pneumonia	13 (19)	1 (20)	9 (32)	1 (4)	2 (22)	0.02 ^5^
Delirium	12 (17)	1 (20)	6 (21)	5 (18)	0 (0)	0.60
Sepsis/SIRS	11 (16)	1 (20)	5 (18)	4 (14)	1 (11)	1
Significant bleeding	10 (14)	0 (0)	7 (25)	3 (11)	0 (0)	0.24
Deep vein thrombosis	6 (9)	1 (20)	2 (7)	3 (11)	0 (0)	0.58
Permanent paraplegia	6 (9)	1 (20)	2 (7)	3 (11)	0 (0)	0.58
ARDS	5 (7)	1 (20)	3 (11)	0 (0)	1 (11)	0.15
Lower limb ischemia	5 (7)	0 (0)	3 (11)	1 (4)	1 (11)	0.69
Major stroke	5 (7)	1 (20)	2 (7)	1 (4)	1 (11)	0.40
Endoleak type IIa	4 (6)	1 (20)	1 (4)	1 (4)	1 (11)	0.51
Visceral infarction	4 (6)	1 (20)	1 (4)	1 (4)	1 (11)	0.32
Pulmonary embolism	3 (4)	0 (0)	2 (7)	1 (4)	0 (0)	1
Transient hemodialysis	3 (4)	0 (0)	1 (4)	2 (7)	0 (0)	1
TIA	2 (3)	0 (0)	0 (0)	1 (4)	1 (11)	0.36
Upper limb ischemia	1 (1)	0 (0)	0 (0)	0 (0)	1 (11)	0.20
In-hospital mortality	4 (6)	0 (0)	2 (7)	1 (4)	1 (11)	0.84
ICU stay duration—d	9 (13)	10 (21)	10 (13)	6.5 (11)	4 (8)	0.91
LOHS—d	15.5 (18)	13 (40)	14.5 (22)	15.5 (15)	19 (7)	0.92

Data are presented as n (%), or median (interquartile range). SIRS = systemic inflammatory response syndrome; ARDS = acute respiratory distress syndrome; TIA = transient ischemic attack; LOHS = length of hospital stay. ^5^ No significance after post hoc tests and Bonferroni correction.

## Data Availability

The data, analytic methods, and study materials will be readily made available upon reasonable request addressed to the corresponding author.

## Abbreviations

The following abbreviations are used in this manuscript:

ARDS	Acute respiratory distress syndrome
ASA	American Society of Anesthesiologists
BMI	Body mass index
BTAI	Blunt traumatic aortic injury
CAD	Coronary artery disease
CHF	Congestive heart failure
COPD	Chronic obstructive pulmonary disease
ESVS	European Society of Vascular Surgery
ICU	Intensive care unit
IQR	Interquartile range
ISS	Injury Severity Score
LOHS	Length of hospital stay
LSA	Left subclavian artery
SD	Standard deviation
SIRS	Systemic inflammatory response syndrome
TEVAR	Thoracic endovascular aortic repair
TIA	Transient ischemic attack
